# Proteomic Epithelial‐To‐Mesenchymal Transition Signature in Fetoplacental Small Extracellular Vesicles of Early‐Onset Preeclampsia

**DOI:** 10.1002/jex2.70122

**Published:** 2026-03-03

**Authors:** Michaela Stoiber, Monika Horvat Mercnik, Birgit Hirschmugl, Barbara Darnhofer, Dominique Pernitsch, Barbara Leopold‐Posch, Ursula Hiden, Dagmar Kolb, Christian Wadsack

**Affiliations:** ^1^ Department of Obstetrics and Gynaecology Medical University of Graz Graz Austria; ^2^ Core Facility Mass Spectrometry Medical University of Graz Graz Austria; ^3^ Core Facility Ultrastructure Analysis Medical University of Graz Graz Austria; ^4^ Research Unit Early Life Determinants Medical University of Graz Graz Austria; ^5^ Gottfried Schatz Research Center for Cell Signaling, Metabolism and Aging Division of Cell Biology, Histology and Embryology Medical University of Graz Graz Austria; ^6^ BioTechMed‐Graz Graz Austria

**Keywords:** extracellular vesicles, foetal development, fetoplacental endothelial cells, human placenta, preeclampsia, proteomics

## Abstract

Preeclampsia (PE), a hypertensive disorder in pregnancy, is linked to placental vascular remodelling, increasing risks of foetal growth restriction and long‐term offspring health problems. The role of fetoplacental endothelial cell‐derived extracellular vesicles (EVs) in PE remains underexplored. This study investigates whether EV composition in Early‐Onset PE (EO‐PE) is altered, potentially contributing to impaired foetal development. Small EVs (sEVs) were isolated from primary fetoplacental endothelial cells (fpECs) of term (T), preterm (PT) and EO‐PE pregnancies. sEVs were characterised using transmission electron microscopy, nanoparticle tracking analysis and Western blotting, confirming spherical morphology, size (<200 nm) and expression of canonical EV and endothelial markers. Proteomic profiling via nano‐LC MS/MS and gene set enrichment analysis revealed a cohesive proteomic profile in fpEC‐derived T‐ and PT‐sEVs, but EO‐PE‐derived sEVs showed heterogeneity and functional alterations compared to T‐ and PT‐derived sEVs. Notably, EO‐PE‐sEVs were enriched in proteins affiliated to epithelial‐to‐mesenchymal transition and myogenesis, processes tied to tissue remodelling and vascular homeostasis, all hallmarks in PE. This signature may represent a molecular signal associated with endothelial dysfunction. In contrast, T‐sEVs were enriched in cell cycle and DNA repair pathways. These findings underscore the role of fetoplacental‐derived EVs in placental‐foetal communication under pathophysiological conditions.

## 1 Introduction

Preeclampsia (PE) is a serious multisystemic pregnancy disorder, affecting around 5%–8% of pregnant women worldwide. It is characterised by the onset of hypertension and dysfunction of end organs (e.g., the placenta, liver and kidney) after 20 weeks of gestation (Brown et al. 2018; Chappell et al. 2021). From a clinical perspective, PE is categorised as either Early‐Onset PE (EO‐PE) or Late‐Onset PE (LO‐PE). EO‐PE is defined as the onset occurring before 34 weeks of gestation, presenting with severe clinical manifestations and worse maternal and foetal outcomes than LO‐PE (Brown et al. 2018; Magee et al. 2022). EO‐PE frequently results from abnormal placentation and impaired placental perfusion, leading to placental hypoxia and inflammation (Dimitriadis et al. 2023). These placental abnormalities not only contribute to maternal systemic endothelial dysfunction, but also compromise foetal health, increasing the risk of intrauterine growth restriction (IUGR), preterm birth (PT) and perinatal morbidity and mortality (Brown et al. 2018; Chappell et al. 2021; Dimitriadis et al. 2023; Magee et al. 2022). In addition to these immediate complications, evidence suggests that exposure to PE in utero can predispose offspring to long‐term health problems, including impaired neurodevelopment (Maher et al. 2018; Tuovinen et al. 2010; Wallace et al. 2008), cardiovascular complications (Andraweera and Lassi 2019; Çetinkaya et al. 2011; Davis et al. 2012; Fugelseth et al. 2011) and immune dysfunction (Kalagiri et al. 2016; Liu et al. 2015; Lodge‐Tulloch et al. 2022; Ye et al. 2025). However, the mechanisms by which PE drive foetal development and subsequently foetal and neonatal health remain unclear.

The placenta is a foetal‐derived organ that serves as vital interface between the maternal and foetal systemic circulations, facilitating nutrient, gas and waste exchange while also modulating maternal immune tolerance and vascular homeostasis (Burton and Jauniaux 2023; Cindrova‐Davies and Sferruzzi‐Perri 2022; Khorami‐Sarvestani et al. 2024; Schliefsteiner et al. 2024). Increasing research highlights extracellular vesicles (EVs), particularly small EVs (sEVs), as key mediators of intercellular communication at the maternal‐foetal interface. Placental EVs carry a complex cargo—proteins, lipids and nucleic acids—that reflect the physiological and pathological state of the placenta and influence the function of recipient cells locally and systemically (David and Maharaj 2025; Escudero and Vatish 2025; Sun et al. 2025).

Structurally, the placenta comprises cellular barriers on both maternal and foetal sides; the syncytiotrophoblasts face maternal blood, while the fetoplacental endothelium lines the foetal vasculature (Burton and Jauniaux 2023; Cindrova‐Davies and Sferruzzi‐Perri 2022; Khorami‐Sarvestani et al. 2024). While most studies to date have focused on trophoblast‐derived EVs, implicating them in maternal immune modulation (Favaro et al. 2021; Mincheva‐Nilsson and Baranov 2014), endothelial activation (Erlandsson et al. 2023; Sha et al. 2023) and systemic inflammation (Germain et al. 2007; Holder et al. 2012), significantly less is known about EVs originating from the foetal‐facing side of the placenta. Given that nearly all viable cells release EVs (Buzas 2023), it is highly plausible that fetoplacental endothelial cells (fpECs) also secrete EVs into the foetal circulation. In particular, the molecular cargo and signalling potential of fpEC‐derived EVs, may point to their role in vascular development, metabolic regulation and immune priming in the foetus. However, this area remains underexplored, particularly in the context of pathological pregnancies. This knowledge gap is especially pertinent in EO‐PE, a condition marked by placental dysfunction, oxidative stress and inflammation. These pathological states are likely to alter the quantity and composition of sEVs secreted by fpECs, potentially affecting foetal development through disrupted signalling pathways. A deeper understanding of the bioactive content of fpEC‐derived sEVs and how their profiles are altered in conditions such as EO‐PE, may uncover novel mechanisms of fetoplacental communication and offer insights into the developmental origins of health and disease.

In this study, we isolated small EVs from primary fpECs obtained from clinically well‐characterised pregnancies, including term (T), preterm (PT) and EO‐PE cases, with gestational age‐matched controls. By leveraging proteomic profiling, we compared sEVs from these well‐selected entities to identify pathophysiological alterations. Our study offers novel insights into the molecular diversity of fetoplacental sEVs, their role in mediating placental‐foetal communication and their potential contribution to foetal development in utero.

## 2 Material and Methods

### 2.1 Sample Collection

After delivery, all placentas were obtained from singleton pregnancies at the Department of Obstetrics and Gynaecology, Medical University of Graz, Graz, Austria. Cohort and sample processing were approved by the ethics committee of the Medical University of Graz (29‐319 ex 16/17), and detailed voluntary consent was obtained from all study participants. Exclusion criteria included maternal systemic diseases, other pregnancy complications, nicotine use during pregnancy and a body mass index of <18 or >27 before pregnancy. The control groups were sampled based on the timepoint of delivery; those delivered at ≥38 weeks of gestation, referred to as term (T) controls, and those delivered before 37 + 0 weeks of gestation, referred to as preterm (PT) controls. The PT group served as an age‐matched control to the Early‐Onset preeclampsia (EO‐PE) group. EO‐PE was diagnosed based on the American College of Obstetricians and Gynaecologists guidelines, defined as new‐onset hypertension with a blood pressure of ≥140/90 mmHg plus organ manifestation, with the onset of maternal symptoms before 34 weeks of gestation (ACOG Board 2020). Detailed characteristics of the study participants are shown in Table 1.

**TABLE 1:** Characteristics of the study cohort.

		Term (*n* = 6)	Preterm (*n* = 6)	Early‐Onset preeclampsia (*n* = 4)	*p* values
Offspring	Gestational days	279 (21)*#	251 (12)*	232.5 (19)#	*0.0143 #0.0002
	Birth weight percentile	25.5 (56)#	54.5 (50)+	9 (24)#+	#0.0464 +0.0039
	Placental weight (g)	670 (350)#	465 (350)+	360 (50)#+	#0.0009 +0.0180
	Foetal sex	2 F; 4 M	4 F; 2 M	3 F; 1 M	
Maternal	Maternal age	28.5 (8)	30 (17)	32.5 (11)	
	BMI before pregnancy	20.25 (4.6)	21.55 (6.1)	21.65 (6.2)	
	sFlt‐1/PlGF ratio	N/A	N/A	372.8 (166.3)	
	Systolic BP (mmHg)	118 (39)#	116 (38)+	174 (36)#+	#0.0050 +0.0042
	Diastolic BP (mmHg)	82 (32)#	72.5 (21)+	103.5 (20)#+	#0.0113 +0.0017
	Uric acid (mg/dL)	N/A	N/A	6.8 (2)	
	CRP (mg/L)	3.6 (7.8)	4.7 (12)	8.65 (38.9)	
	Urinary protein	N/A (dipstick negative)	N/A (dipstick negative)	327 (597) (*n* = 3)	

*Note*: Data is represented as median (range). Kruskal–Wallis test + Dunn's post hoc test was performed to observe the statistical difference between the groups *Term (T) versus Preterm (PT), #T versus Early‐Onset preeclampsia (EO‐PE), and +PT versus EO‐PE.

Abbreviations: BMI, body mass index; BP, blood pressure; CRP, C‐reactive protein; F, female; M, male; N/A, not available; PlGF, placental growth factor; sFlt‐1, soluble fms‐like tyrosine kinase‐1.

### 2.2 Isolation and Cultivation of Primary Fetoplacental Endothelial Cells (fpECs)

Isolation and cultivation of fpECs were performed as previously described (Allerkamp et al. 2025; Lang et al. 2008). In summary, arteries were excised from the chorionic plate of the placenta and rinsed with Hank's balanced salt solution (HBSS; Gibco, Thermo Fisher Scientific, Waltham, MA). fpECs were isolated by perfusion of the arteries with 20 mL of prewarmed HBSS containing 0.1 U/mL collagenase, 0.8 U/mL dispase II (Roche, Basel, Switzerland) and 10 mg/mL penicillin/streptomycin (Gibco) at a flow rate of 2.5 mL/min. Cells were collected in 10 mL foetal calf serum (FCS, Gibco) and centrifuged at room temperature at 900 rpm for 7 min. The cell pellet was resuspended in Endothelial Cell Growth Medium MV (ECGM; PromoCell, C‐22220, Heidelberg, Germany), supplemented with endothelial cell growth factor, hydrocortisone, epidermal growth factor (PromoCell, C‐39220), 0.05 mg/mL gentamicin (Gibco) and 10% defined FCS and plated in 12‐well plates coated with 1% gelatine (Sigma–Aldrich, St. Louis, MO). The cells were incubated at 37°C in 12% O_2_ and 5% CO_2_. Upon reaching confluence, the cells were transferred to cell culture flasks, maintained under the same conditions, frozen and stored in liquid nitrogen until further use. The identity and purity of fpECs were confirmed by flow cytometry as reported in Allerkamp et al. (2025).

### 2.3 Isolation of Small Extracellular Vesicles (sEVs)

fpECs were thawed and expanded. Prior to sEVs isolation, cells (7th to 11th passage) were seeded at a density of 1.5 × 10^6^ cells per 175 cm^2^ flask and cultured in ECGM to 80% confluency. fpECs were washed twice with HBSS and cultured for 48 h in sEV‐depleted medium (25 mL/175 cm^2^ flask, Endothelial Cell Growth Medium MV media supplemented with 0.05 mL/mL exosome‐depleted FCS (Gibco, #A2720801), together with 10 ng/mL recombinant human epidermal growth, 1 µg/mL hydrocortisone and 0.001 mL/ml Gentamycin. Upon isolation, the cell number was counted using CASY cell counter (OMNI Life Science), and conditioned media (CM) were used for the enrichment of sEVs with an adapted protocol by Momen‐Heravi (Momen‐Heravi 2017). CM (25 mL/175 cm^2^ flask) was collected and centrifuged at 500 × *g* for 10 min at 4°C to remove detached cells. Again, supernatants were centrifuged at 2500 × *g* for 20 min at 4°C to remove cell debris and apoptotic bodies. To remove large extracellular vesicles (lEVs), the supernatant was centrifuged at 12,000 × *g* for 45 min at 4°C (Optima XE‐90, Type 70 Ti rotor, with Ultra‐Clear Centrifuge Tubes, 25 × 89 mm, 38.5 mL, Beckman Coulter #344058). The pellet was discarded and the pooled supernatants were filtered through a 0.22 µm filter (Millipore, #SCGP00525).

A protein concentrator (Pierce Protein Concentrator PES, 100,000 MWCO, 20–100 mL, ThermoFischer Scientific, Waltham, MA) was used to reduce the volume (25 mL CM to approx. 4 mL) at 1800 × *g* for 15 min at 4°C. Subsequently, the concentrated CM was centrifuged at 100,000 × *g* for 2 h at 4°C (Optima XE‐90, Type 70 TiRotor, with Quick‐Seal, 12.5 mL, Beckman Coulter #342413). The pellet was washed once with 0.02 µm‐filtered PBS under the same centrifugation conditions (100,000 *g* for 2 h at 4°C) and resuspended either in 250 µL of 0.02 µm‐filtered PBS for NTA and TEM or in 100 µL RIPA buffer for proteomic analysis or Western blot. All sEVs enriched solutions were stored in microcentrifuge tubes (Low Protein Binding, ThermoFisher, #90410) at –80°C until further processing.

### 2.4 Nanoparticle Tracking Analysis (NTA)

sEVs were isolated from one 175 cm^2^ flask (25 mL CM) from T‐fpECs (*n* = 6), PT‐fpECs (*n* = 6) and EO‐PE‐fpECs (*n* = 4). sEVs size distribution and particle concentration were determined by nanoparticle tracking analysis (NTA) using NanoSight NS300 (Malvern Panalytical GmbH, Kassel, Germany), equipped with a 405 nm laser module and software version 3.3. Samples were diluted (1:20 for T‐sEVs, 1:50 in PT‐ and EO‐PE‐sEVs) in a total volume of 1500 µL in filtered PBS as recommended by the manufacturer. Capture and analysis settings were set as following: Syringe pump speed: 50, Camera Level: 12, Slider Shutter: 1200, Slider Gain: 146, Detection Threshold: 3. Five videos (technical replicates) of 60 s each were captured to obtain the mean and mode particle size and concentration of particles at the size of 0–1000 nm.

### 2.5 Transmission Electron Microscopy (TEM) Analysis

sEVs were harvested from the CM of one 175 cm^2^ flask (25 mL CM) of T‐, PT‐ and EO‐PE‐fpECs (*n* = 1 per group) and resuspended in 250 µL PBS. Prior to grid preparation, sEVs were diluted 1:2 to 1:5 in PBS. Carbon‐coated copper grids were glow‐discharged to render the surface hydrophilic. Grids were floated on a droplet of diluted sEV suspension for 1 min, and excess liquid was subsequently blotted off with filter paper. The grids were then incubated on a droplet of 1% uranyl acetate for 1 min for negative staining. After staining, excess solution was removed and the grids were air‐dried.

Electron micrographs were acquired using a Tecnai G2 transmission electron microscope (FEI, Eindhoven, the Netherlands) equipped with a Gatan Rio 16 high‐speed CMOS camera, operating at an acceleration voltage of 120 kV.

### 2.6 Western Blot Analysis

sEVs were harvested from CM of one 175 cm^2^ flask (25 mL CM) from T‐ PT‐ and EO‐PE‐fpECs (*n* = 1 per group) and resuspended in 100 µL RIPA buffer. The protein concentration was determined using the Micro BCA Protein Assay Kit (Thermo Fisher, #23235). A total of 0.5 µg of protein was diluted in 4× Laemmli buffer (Bio‐Rad, Hercules, USA) and heated at 95°C for 5 min before being loaded onto a 4%–20% Tris‐Glycine gradient gel (Mini‐PROTEAN TGX Precast Protein Gels, Bio‐Rad) for electrophoresis. Proteins were then transferred onto a Nitrocellulose membrane (Trans‐Blot Turbo Mini Nitrocellulose, Bio‐Rad).

The membranes were blocked with 5% BSA (Sigma, A2153‐100G) in Tris/Borate/EDTA (TBE, Gatt‐Koller, Absam, Austria) buffer containing 0.1% Tween 20 (Merck, Darmstadt, Germany) for 1 h at room temperature. Subsequently, membranes were subjected to different primary antibodies: Syntenin‐1 (1:750, Abcam, ab19903), TSG101 (1:1000, Abcam, ab83), Alix (1:1000, CovaLab, pab0204), CD81 (1:500, GeneTex, GTX101766), CD31 (1:1000, Abcam, ab9498) and Apolipoprotein B (1:1000, Abcam, ab39560). After incubation at 4°C overnight, membranes were treated with horseradish peroxidase (HRP)‐conjugated secondary antibodies anti‐mouse (1:2000, BIO‐RAD, 170–6526) or anti‐rabbit (1:2000, BIO‐RAD, 170–6525) for 1 h. Detection was performed using SuperSignal West Pico substrate (Bio‐Rad, Hercules, CA, USA) and signals were visualised with the Fusion Fx Imaging System (Vilber Lourmat, Collégien, France).

### 2.7 Mass Spectrometric Analysis

sEVs were isolated from CM (25 mL) of one 175 cm^2^ flask from T‐fpECs (*n* = 4), PT‐fpECs (*n* = 5) and EO‐PE‐fpECs (*n* = 4) and resuspended in 100 µL RIPA buffer. Aliquots from pooled CM were analysed by both, NTA and Mass Spectrometry, which related the number of particles to the total protein of each condition. Total protein concentration was determined using the Micro BCA Protein Assay Kit (Thermo Fisher, #23235). For Liquid chromatography‐mass spectrometry/mass spectrometry (LC‐MS/MS) analysis 5 µg of total protein per sample were reduced and alkylated for 40 min at 60°C with final 10 mM tris(2‐carboxyethyl)phosphine and 40 mM 2‐Chloroacetamide. All samples were processed according to the SP3 protocol (Hughes et al. 2019) and digested overnight with trypsin (Promega, enzyme/protein 1:50). Peptides were desalted using SBD‐RPS tips as previously published (Darnhofer et al. 2021). A 400 ng per sample (re‐dissolved in 2% acetonitrile/0.1% formic acid in water) was subjected to LC‐MS/MS analysis. Protein digests were separated by nano‐HPLC (Dionex Ultimate 3000, Thermo Fisher Scientific) equipped with a C18, 5 µm, 100 Å, 100 µm × 2 cm enrichment column (Thermo Fisher Scientific, Austria) and an Acclaim PepMap RSLC nanocolumn (C18, 2 µm, 100 Å, 500 × 0.075 mm, Thermo Fisher Scientific, Vienna, Austria). Samples were concentrated on the enrichment column for 5 min at a flow rate of 15 µL/min with 0.1% formic acid as an isocratic solvent. Separation was carried out on the nanocolumn at a flow rate of 300 nL/min at 60°C using the following gradient, where solvent A is 0.1% formic acid in water and solvent B is acetonitrile containing 0.1% formic acid: 0–5 min: 2% B; 5–123 min: 2%–35% B; 123–124 min: 35%–95% B, 124–134 min: 95% B; 134–135 min: 2% B; 135–150 min: 2% B. The maXis II ETD mass spectrometer (Bruker Daltonics, Germany) was operated with the captive source in positive mode with the following settings: mass range: 200–2000 m/z, 2 Hz, capillary 1600 V, dry gas flow 3 L/min with 150°C, nanoBooster 0.2 bar, precursor acquisition control top 20 (collision induced dissociation (CID). The mass spectrometry proteomics data were deposited to the ProteomeXchange Consortium (Vizcaíno et al. 2014) via the partner repository with the dataset identifier PXD068733 (Vizcaíno et al. 2014).

## 3 Data Analysis

### 3.1 Clinical Data

Statistical analyses were performed using R version 4.4.1 and RStudio. The Kruskal‐Wallis test followed by Dunn's post hoc test using the R package dunn.test (Dinno 2024) was conducted to assess statistical differences between groups.

### 3.2 NTA Data Analysis

Statistical analysis was performed using one‐way ANOVA followed by Tukey's HSD post hoc test for multiple comparisons using the R packages stats (R Core Team 2024), car (Fox and Weisberg 2019) and multcomp (Hothorn et al. 2008).

### 3.3 TEM Data Analysis

Recorded TEM images were analysed using FIJI (Image J version 1,53t). Round particles exhibiting a characteristic darker electron dense rim were considered sEVs. Particle diameters were measured using the ‘Measure’ function (*n* = 50 sEVs per group).

### 3.4 Proteomics Data Analysis

The LC‐MS/MS data were analysed by MaxQuant by searching the public SwissProt human database (11393515 residues, 20467 sequences) and common contaminants (Tyanova et al. 2016). Carbamidomethylation on cysteine and oxidation on methionine were set as fixed and variable modifications, respectively. Detailed search criteria were used as follows: trypsin, max. missed cleavage sites: 2; search mode: MS/MS ion search with decoy database search included; precursor mass tolerance ± 10 ppm; product mass tolerance ± 25 ppm; acceptance parameters for identification: 1% PSM FDR; 1% protein FDR. Additionally, a label free quantification (LFQ) was performed using MaxQuant, requiring a minimum of two ratio counts of quantified razor and unique peptides.

Data processing was performed using Perseus software version 2.0.10.0. The data was filtered for decoy hits, contaminants and proteins identified only by modified peptides. After performing a log_2_ transformation, proteins were filtered to include only those with at least 75% valid values per group. Missing values were imputed with random values drawn from a Gaussian distribution. A two‐sided *t*‐test was conducted with *p* values corrected for false discovery rate (FDR), and proteins were considered statistically significant if they had an absolute log_2_ fold change (log_2_FC) ≥ 1.2 and a *p* value ≤ 0.05. Additionally, an unsupervised Gene Set Enrichment Analysis (GSEA, version 4.4.3) was performed to assess the biological relevance of the sEVs proteome. The following R packages were utilised for visualisation and statistical analysis: stats (R Core Team 2024), ggplot2 (Wickham 2016), VennDiagram (Chen and Boutros 2011) and ComplexHeatmap (Gu 2022; Gu et al. 2016).

## 4 Results

### 4.1 Characterisation of sEVs Isolated From Fetoplacental Primary Endothelial Cells (fpEC)

First, sEVs released by fpECs derived from placentas of T‐, PT‐ and EO‐PE‐pregnancies were comprehensively characterised in accordance with the MISEV23 guidelines (Welsh et al. 2024).

**FIGURE 1:**
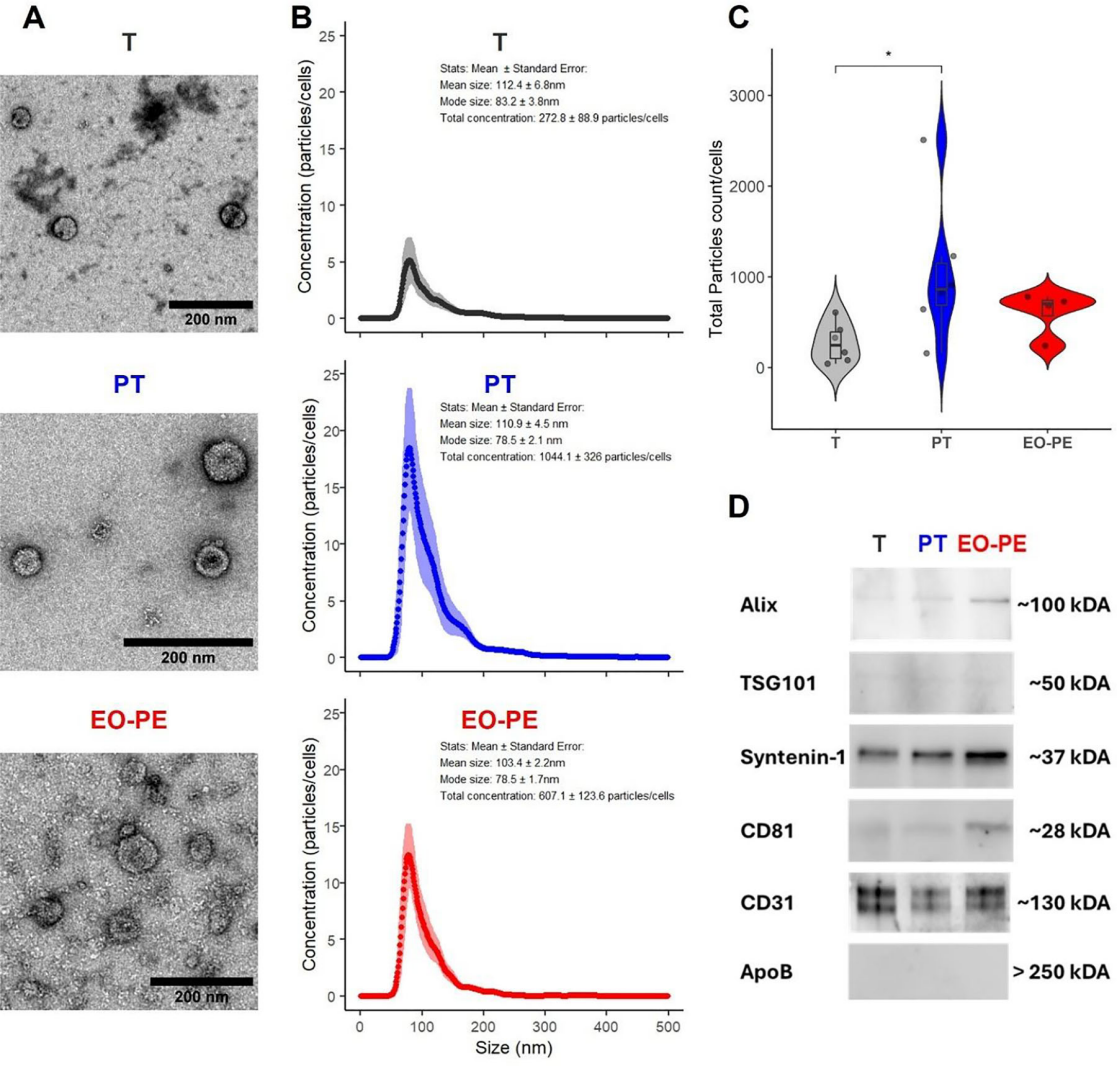
Characterisation of sEVs secreted by fetoplacental endothelial cells (fpECs) from term (T, *n* = 6), preterm (PT, *n* = 6) and Early‐Onset preeclamptic (EO‐PE, *n* = 4) pregnancies. (A) Representative transmission electron microscopy (TEM) images of sEVs (scale bar: 200 nm). (B) NTA: Size and concentration of particles, with size (x‐axis) plotted against particle concentration normalised to cell count at the time of isolation (y‐axis). Data are presented as mean ± standard error (SE). (C) Violin plots display the total concentration of sEVs (normalised to cell count at isolation) as measured by NTA. Statistical analysis was performed using the Kruskal–Wallis test, followed by pairwise Wilcoxon rank‐sum tests with Bonferroni correction for multiple comparisons. Adjusted *p* values are indicated (* = *p* < 0.05; ns = not significant). (D) Western blot analysis of sEVs using established sEV markers (Alix, TSG101, Syntenin‐1), endothelial marker (CD31) and non‐vesicular marker (ApoB).

Transmission electron microscopy (TEM) images show the presence of sEVs in all three groups (Figure 1A). The vesicles display the typical round‐shaped membrane‐enclosed morphology with variable electron density and sizes consistent with sEVs (50–150 nm). To quantify size heterogeneity, a quantitative analysis of TEM images (*n* = 50 vesicles per group) revealed mean diameters of 65.6 ± 44.1 nm for T‐sEVs (range: 22.5–296.2 nm), 66.8 ± 32.4 nm for PT‐sEVs (range: 29.1–166.9 nm) and 44.6 ± 14.4 nm for EO‐PE‐sEVs (range: 21.8–75.0 nm) (Figure S1A).

Nanoparticle tracking analysis (NTA) was used to assess particle size and concentration in suspension. Mean particle sizes (± standard error) were 112.4 ± 6.8 nm for T‐sEVs, 110.9 ± 4.5 nm for PT‐sEVs and 103.4 ± 2.2 nm for EO‐PE‐sEVs. Mode particle sizes (± standard error) were 83.2 ± 3.8 nm for, 78.5 ± 2.1 nm and 78.5 ± 1.7 nm for T‐, PT‐ and EO‐PE‐sEVs, respectively (Figure 1B, Figure S1B,C). The smaller sizes observed by TEM likely reflect vesicle shrinkage during dehydration, whereas NTA measures hydrodynamic diameter in solution, resulting in larger size estimates.

To evaluate whether placentas from different entities may influence sEV abundance, the number of particles (as a surrogate for sEVs) were normalised to the cell number at the time of collecting media supernatants. The mean concentrations were 272.8 ± 88.9, 1044.1 ± 326 and 607.1 ± 123.6 particles/cell in T, PT and EO‐PE groups, respectively. This approximate analysis showed a significant increase of particles per cell, in the PT group compared to the T group (Figure 1C, *P* = 0.025, Kruskal–Wallis and pairwise Wilcoxon rank‐sum tests with Bonferroni correction).

Western blot analysis demonstrated the presence of classical EV markers Alix, TSG101, Syntenin‐1 and CD81 across all groups, indicating successful sEV enrichment (Figure 1D). The endothelial marker CD31 was also detected, verifying the endothelial origin of the vesicles. Importantly, Apolipoprotein B (ApoB), a marker for larger lipoproteins, was absent in all samples, supporting the high purity of isolates. The presence of EV markers (Alix, TSG101, Syntenin‐1 and CD81) and endothelial markers (CD31, VWF) was further supported by proteomic analysis (Figure S1B).

### 4.2 Proteomic Profiling and Differential Abundance Analysis of T‐, PT‐ and EO‐PE‐Derived sEVs

sEVs are key mediators of intercellular communication and are known to influence the metabolic and functional state of recipient cells. As such, their protein cargo represents a source of biologically informative molecules and provides an opportunity for comparative proteomic analysis across (patho)‐physiological conditions. To assess the protein composition of T‐, PT‐ and EO‐PE‐sEVs, nano‐LC‐MS/MS analysis was performed.

**FIGURE 2:**
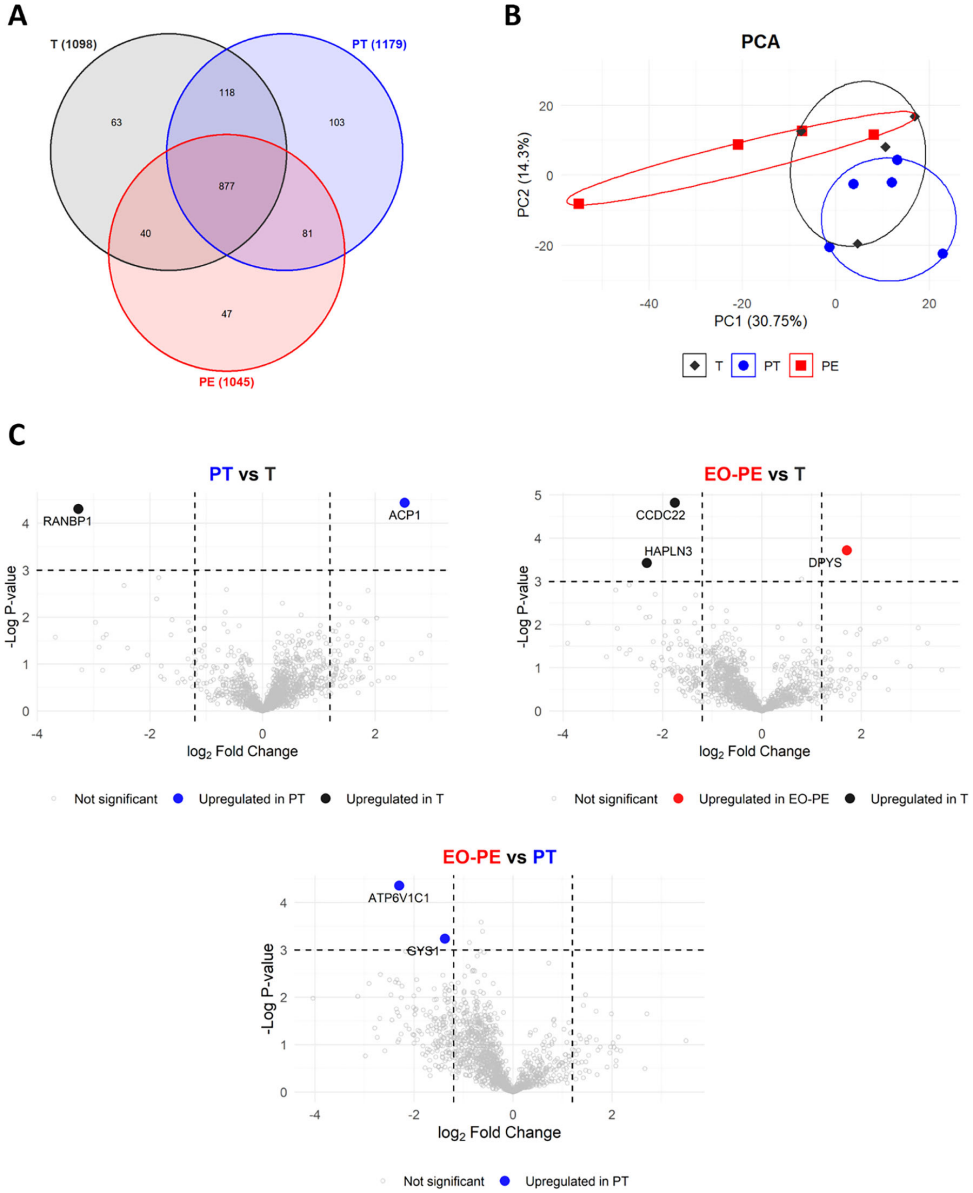
Descriptive statistics of proteomic analysis of fetoplacental‐endothelial derived sEVs from T (*n* = 4), PT (*n* = 5) and EO‐PE (*n* = 4) pregnancies. (A) Venn diagram of identified proteins in the respective groups. Proteins were filtered to include those with >75% valid values in each group, resulting in 1098 proteins identified in T‐sEVs, 1179 proteins in PT‐sEVs and 1045 proteins in EO‐PE‐sEVs. (B) Principal component analysis (PCA) of protein abundances in the proteome. PCA revealed distinct clustering between the proteomes of EO‐PE‐ and PT‐sEVs, while T‐sEVs overlapped both, indicating shared variability. (C) Volcano plots of quantified proteins. Significant proteins are highlighted in dark grey (T), blue (PT) and red (EO‐PE) circles (two‐sided *t*‐test with *p* values corrected for FDR, statistically significant if absolute log_2_FC ≥ 1.2 and *p* value ≤ 0.05), while non‐significant proteins are depicted in smaller grey circles.

To ensure robustness of obtained data, proteins identified in ≥75% of samples within a group (*n* = 4–6) were considered representative of that group. Using this threshold, a total of 1329 unique proteins were detected across all groups (Figure 2A). The highest number of proteins was found on PT‐sEVs (1179), followed by T‐sEVs (1098) and EO‐PE‐sEVs (1045). A core set of 877 proteins (66.0%) was shared among all three groups, indicating substantial overlap in the fpECs‐derived sEV proteome. PT‐sEVs also exhibited the highest number of unique proteins (103; 7.8%), followed by T‐sEVs (63; 4.7%) and EO‐PE‐sEVs (47; 3.5%). In addition, 118 proteins (8.9%) were shared between T and PT, 61 proteins (4.6%) between PT and EO‐PE and 40 proteins (3.0%) between T and EO‐PE.

PCA analysis (Figure 2B) was performed to assess global differences in proteomic profiles of sEVs. The first two components, PC1 and PC2, accounted for 30.7% and 14.3% of the total variance, respectively. T and PT samples clustered closely together, indicating high similarity, while EO‐PE samples displayed broader dispersion, reflecting greater heterogeneity within this group. Importantly, PT and EO‐PE groups formed clearly distinct, non‐overlapping clusters, indicating significant proteomic divergence. In contrast, T and EO‐PE groups partially overlap, suggesting the presence of shared proteomic features, whereas T and PT profiles remained largely overlapping.

To identify differentially abundant proteins, differential protein abundance analysis was conducted across the following group comparisons: T versus PT, T versus EO‐PE and PT versus EO‐PE (Figure 2C). Proteins were considered significantly differentially abundant if they exhibited an absolute log_2_ fold change (log_2_FC) ≥ 1.2 and an adjusted *p* value ≤ 0.05.

In the PT versus T comparison, RANBP1, involved in Ras‐related protein transport and cell cycle regulation, was significantly more abundant in T‐sEVs (log_2_FC = –3.272, adj. *p* = 0.013). In contrast, ACP1, an acid and tyrosine phosphatase, was elevated in PT‐sEVs (log_2_FC = 2.523, adj. *p* = 0.012). In the EO‐PE versus T comparison, CCDC22 (log_2_FC = –1.752, adj. *p* = 0.008), a protein involved in NF‐κB signalling and HAPLN3 (log_2_FC = –2.315, adj. *p* = 0.032), a cell adhesion molecule, were more abundant in T‐sEVs. Conversely, DPYS, an enzyme involved in pyrimidine metabolism, was increased in EO‐PE‐sEVs (log_2_FC = 1.707, adj. *p* = 0.024). Finally, comparison of EO‐PE versus PT, revealed increased levels of ATP6V1C1 (log_2_FC = –2.300, adj. *p* = 0.013), a subunit of the vacuolar ATPase complex critical for intracellular pH regulation and protein trafficking and GYS1 (log_2_FC = 1.370, adj. *p* = 0.039), a key enzyme in glycogen biosynthesis upregulated in PT‐sEVs.

### 4.3 EO‐PE‐sEVs Are Enriched in Epithelial‐to‐Mesenchymal Transition (EMT) Proteins

**FIGURE 3:**
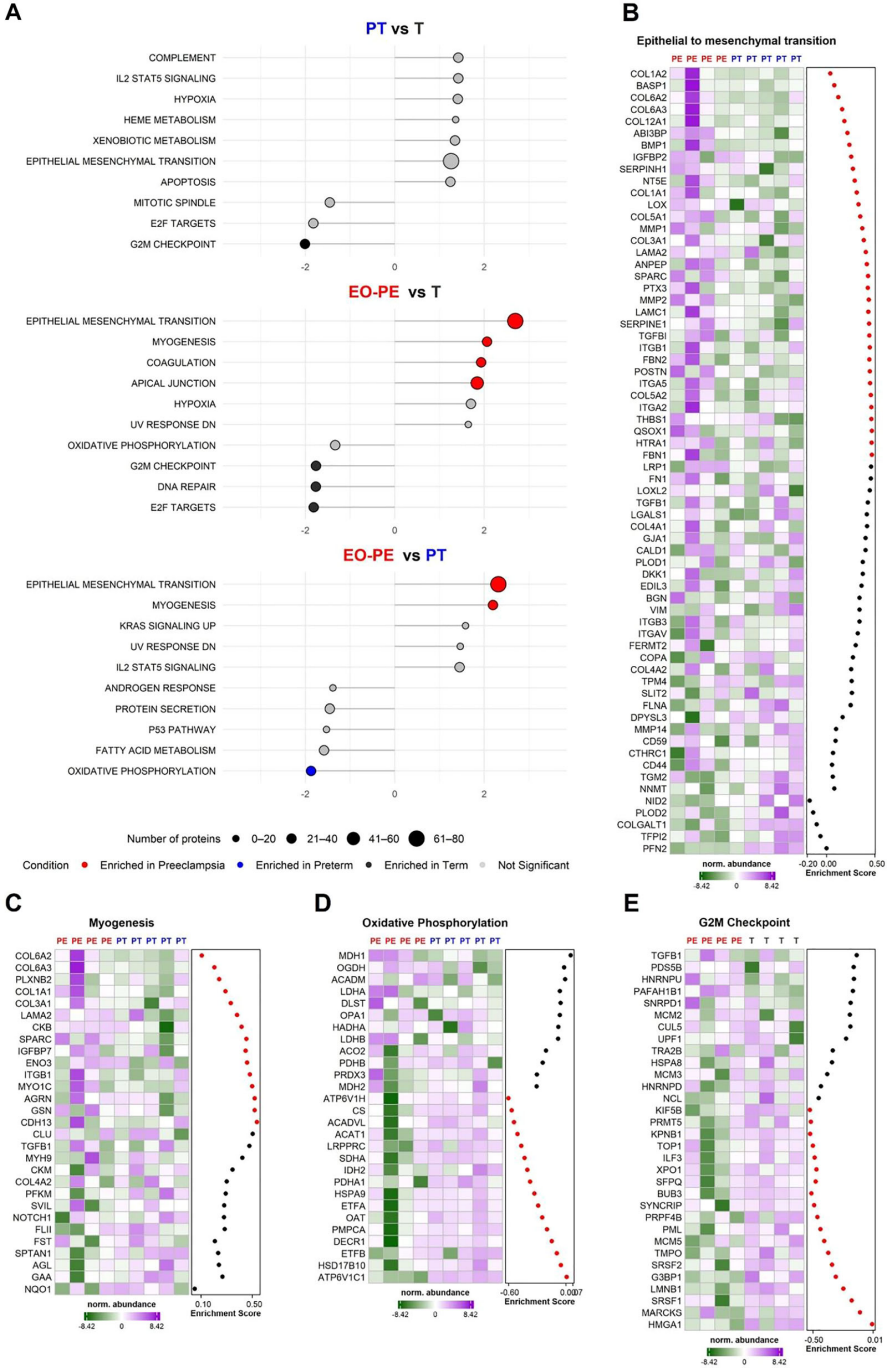
Unsupervised GSEA and hallmark‐specific protein abundance across fp‐EC derived sEVs in T, PT and EO‐PE pregnancies. (A) GSEA was performed for the comparisons PT versus T, EO‐PE versus T and EO‐PE versus PT using the log_2_‐transformed LFQ‐values from all detected proteins in the sEVs using the Hallmark gene sets from the Molecular Signature Database (MSigDB). The lollipop‐plot displays the normalised enrichment score (NES; lollipop length), with dot size representing the number of detected proteins detected per pathway. Dot colour indicates the condition in which the pathway is significantly enriched (nominal *p* < 0.01), T (black), PT (blue), EO‐PE (red) or not significant (grey). Top 10 gene sets by NES—regardless of significance‐ are shown for each comparison. Heatmaps show the normalised abundance of individual proteins within the representative enriched pathways in (B) epithelial‐to‐mesenchymal transition (EMT, EO‐PE vs. PT), (C) myogenesis (EO‐PE vs. PT) and (D) oxidative phosphorylation (EO‐PE vs. PT) and (E) G2M Checkpoint (EO‐PE vs. T). Each row represents a protein, ranked by its contribution to pathway enrichment. The enrichment score for each protein is shown to the right of each heatmap. Core‐enriched proteins are highlighted in red, while non‐core‐enriched proteins are shown in black.

To investigate the functional relevance of the sEV proteome, an unsupervised gene set enrichment analysis (GSEA) was performed using log_2_‐transformed label‐free quantification (LFQ) values for all detected proteins (based on our stringent filtering), based on the Hallmark gene sets from the Molecular Signatures Database (MSigDB). Comparative analyses were conducted for PT versus T, EO‐PE versus T and EO‐PE versus PT (Figure 3A).

Across all comparisons, GSEA consistently revealed significant enrichment of specific hallmark pathways in EO‐PE‐derived sEVs (Figure 3A, middle and lower panels). Notably, epithelial‐to‐mesenchymal transition (EMT, Normalised Enrichment Score (NES) = 2.69 vs. T; NES = 2.31 vs. PT) and myogenesis (NES = 2.06 vs. T; NES = 2.19 vs. PT) gene sets were robustly enriched in EO‐PE‐sEVs. Core enriched proteins driving the EMT signature included multiple collagens (e.g., COL6A2, COL1A1, COL12A1), extracellular matrix (ECM) remodellers (LOX, SPARC, BMP1, MMP1) and growth factors such as IGFBP2, as illustrated in the heatmap (Figure 3B, EO‐PE vs. PT comparison). These proteins were similarly enriched in the EO‐PE versus T comparison (Figure S2A). Proteins contributing to the myogenesis hallmark—such as collagens and myogenic regulators LAMA2 and MYO1C—were also elevated. (Figure 3C, EO‐PE vs. PT comparison; Figure S2B, EO‐PE vs. T comparison). Furthermore, EO‐PE‐sEVs were enriched for protein sets related to apical junction and coagulation (Figure 3A, middle panel; Figure S2C,D). Collectively, these findings suggest that EO‐PE‐sEVs carry a distinct proteomic signature reflecting dysregulated ECM remodelling and vascular homeostasis, consistent the pathophysiology of PE.

In contrast, a unique enrichment pattern associated with cell cycle regulation and genomic stability was observed in T‐sEVs. Specifically, the G2/M checkpoint was enriched in T‐sEVs compared to both EO‐PE (NES = –1.76) and PT (NES = –2.09) (Figure 3A, upper and middle panels). Contributing proteins included HMGA1, MARCKS, SRSF1 and LMNB1, with a more prominent enrichment observed in the EO‐PE versus T comparison (Figure 3E) than in PT versus T (Figure S2G). Additionally, the E2F targets and DNA repair proteins were enriched in T‐sEVs relative to EO‐PE‐sEVs (Figure S2E,F). These findings may reflect the influence of gestational age on proliferative signalling and genome maintenance, with more pronounced dysregulation observed in EO‐PE.

Although PT‐sEVs overall exhibited a proteomic profile closely resembling that of T‐sEVs, selective enrichment of the oxidative phosphorylation hallmark was observed in PT‐sEVs compared to EO‐PE‐sEVs (NES = –1.84) (Figure 3A, lower panel). Core enriched proteins included DECR1, ETFB, HSD17B10 and ATP6V1C1 (Figure 3D), the latter which was also identified as significantly upregulated in PT‐sEVs in the differential protein abundance analysis (Figure 2C). This enrichment suggests that PT‐sEVs may carry a distinct mitochondrial metabolic signature. Given that impaired oxidative phosphorylation has been implicated in the pathogenesis of PE, this finding may reflect differential mitochondrial function between PT and EO‐PE conditions.

#### 4.3.1 Distinct Role of EO‐PE‐sEVs on Target Cells

To test whether EMT‐ and ECM‐related protein signatures in EO‐PE–derived sEVs are associated with endothelial responses, primary human umbilical vein endothelial cells (HUVECs) were used as a target model. HUVECs were treated for 48 h with sEVs from T‐, PT‐ and EO‐PE‐fpECs. This experimental setup allowed assessment of transcriptional and morphological changes in endothelial cells, in line with the enrichment of ECM‐associated proteins in EO‐PE‐sEVs, such as collagens, LOX, SPARC, MMPs and LAMA2 (Figure 3B).

RT‐qPCR analysis revealed a significant downregulation of CDH5 (vascular endothelial cadherin) in HUVECs treated with PT‐ (*p* = 0.028) and EO‐PE‐ (*p* = 0.038) derived sEVs, compared to untreated controls, consistent with alternations in endothelial junction‐associated gene expression. In contrast, the expression of mesenchymal and EMT‐associated genes remained largely unchanged across all treatment conditions. Overall, the endothelial phenotype was preserved, with increased vWF expression observed in T‐sEVs treated HUVECs (*p* = 0.031), but differed significantly between the PT‐ and EO‐PE‐sEV groups (*p* = 0.031) (Figure S3A). As EMT is typically accompanied by morphological changes, we assessed HUVEC morphology by brightfield microscopy. HUVECs formed a confluent monolayer, with typical cobblestone morphology. T‐sEVs treated cells appeared denser and more uniform, showing relatively continuous cell to cell borders. PT‐ and EO‐PE‐sEV treated cells remained largely cobblestone and confluent, but displayed pronounced heterogeneity in density and some elongated cells with overt monolayer disruption. These early signs in cell culture reveal subtle alternations in endothelial cell morphology and monolayer organisation induced by PT‐ and EO‐PE‐derived sEVs (Figure S3B).

## 5 Discussion

Although extensive research has demonstrated the potential of placental sEVs as mediators of intercellular communication between mother and foetus, much remains to be explored to substantiate this concept. In particular, the role of large biomolecules on sEVs, such as proteins, under non‐physiological pregnancy conditions is underexplored. To our knowledge this is the first study to characterise sEVs from primary fetoplacental endothelial cells (fpECs) across term (T), preterm (PT) and Early‐Onset preeclampsia (EO‐PE) pregnancies, controlling for gestational age effects. The secretion and cargo composition of sEVs change dynamically across gestation in normal pregnancies (Salomon et al. 2014). It is also known that EO‐PE and LO‐PE represent clinically distinct entities with different long‐term consequences on the mother and foetus, which is another level of complexity for sEV characterisation (Wadhwani et al. 2020). Therefore, it is important to emphasise that this study focuses on isolated sEVs of EO‐PE cases only, and that the results should not be extrapolated to other pathophysiological types of PE. Proteomic analysis revealed substantial cargo overlap among the three groups. Notably, gene set enrichment analysis (GSEA) identified distinct enrichment of proteins affiliated to epithelial‐to‐mesenchymal transition (EMT) and myogenesis hallmark pathways in EO‐PE‐sEVs. In contrast, T‐sEVs were enriched of proteins involved in cell cycle control‐ and DNA repair mechanisms. These findings suggest that, when released into the extracellular space, fetoplacental sEVs possess unique cargo compositions and protein‐loading capacities. They may therefore act as signalling entities that reflect the (patho)physiological state of their tissue of origin.

Our study identified differences in the number of processed sEVs per cell in PT‐ and EO‐PE—fpECs compared to T—fpECs. Alongside the distinct protein loading capacity of sEVs, these results suggest that the metabolic condition of parental cells influences the quantity and composition of released sEVs. However, measurements of sEV abundance, even with optimal experimental design, represent only a snapshot under normalised cell culture conditions. Placental sEVs are present in foetal blood, with a subset carrying placental‐type alkaline phosphatase (PLAP), a placenta specific marker, indicating their placental origin (Miranda et al. 2018). However, PLAP expression on EVs from the foetal side of the placenta is debated (Kumar et al. 2025; Miranda et al. 2018). Notably, our proteomics analysis revealed the absence of PLAP in fpEC‐derived sEVs. Previous studies on cord plasma reported no differences in total EV levels between PT and T pregnancies (Menon et al. 2022; Murphy et al. 2025), or between PE and T pregnancies (Jia et al. 2015). However, these studies did not specifically examine placental EV subsets, which highlights the importance of studying defined sEV‐populations by addressing their source, such as fpEC‐derived sEVs. While bulk cord plasma analyses showed stable EV concentrations across conditions, isolated fpEC‐derived sEVs reveal pregnancy specific differences that would otherwise remain undetected.

An ex vivo placental perfusion approach showed no differences in the concentration of syncytiotrophoblast‐derived EVs between T and PE pregnancies. (Cronqvist et al. 2017; Erlandsson et al. 2023), suggesting similar EV release by the placenta, regardless of pregnancy condition. In contrast, studies of PLAP^+^ EVs in maternal plasma, reported elevated levels in PE (Rao et al. 2024; Salomon et al. 2017). These PLAP^+^ EVs carry pro‐inflammatory and anti‐angiogenic mediators, supporting the hypothesis that ‘pro‐inflammatory’ sEVs contribute to PE pathophysiology(Goswami et al. 2006; Pillay et al. 2016, 2019). Compared to T pregnancies, PT pregnancies showed lower PLAP^+^ EVs levels in maternal plasma (Menon et al. 2020), suggesting distinct mechanisms of EV release and clearance across pregnancy conditions. These measurements reflect EV concentrations at the time of sampling, but most studies do not distinguish between release, uptake or clearance dynamics, which may be influenced by vesicle cargo and interactions with target cells. Collectively, these findings, including ours, indicate a complex control mechanism for sEV secretion, influenced by diverse conditions, dual circulations and various cell types in the human placenta. However, no direct mechanism has been established linking altered EV concentrations contribute to PE pathology.

Proteomic profiling revealed 66% of shared proteins across all three groups, supporting a conserved vesicular proteome in fpECs‐derived sEVs. However, when quantitative abundance levels were considered, EO‐PE‐derived sEVs exhibited heterogeneity of proteins and more pronounced functional divergences compared to T‐ and PT‐derived sEVs.

Importantly, the increased heterogeneity of EO‐PE‐sEVs is unlikely to result from technical variation, as fpEC isolation, cell culture, sEV preparation and LC‐MS/MS analysis were identical across all groups, and quality control showed no systematic differences. Instead, this heterogeneity is consistent with previously published bulk RNA‐sequencing data from the same fpEC isolates (Allerkamp et al. 2025), which revealed substantial transcriptional diversity in EO‐PE–derived endothelial cells. Therefore, the proteomic variability of EO‐PE‐sEVs likely reflects true biological heterogeneity rather than technical noise.

Observed differences were not driven by unique or highly abundant protein differences, but by quantitative shifts among proteins with similar functions. To explore this, we analysed the entire set of identified proteins with their respective LFQ values, focusing on functional patterns arising from these shifts. EO‐PE‐derived sEVs were enriched in proteins associated with tissue remodelling and vascular adaptations, while T‐derived EVs are enriched in cell cycle control proteins. Consistent with our results, PE‐derived cord plasma EVs showed enrichment in proteins linked to coagulation pathway alterations (Jia et al. 2015) and similar findings were reported in maternal serum (Youssef et al. 2021). Subtle proteome differences were observed between T‐ and PT‐derived sEVs, aligning with prior studies reporting minor differences in PT‐derived cord plasma EVs to T (Menon et al. 2022). These findings demonstrate, that EO‐PE‐sEVs exhibit broader functional heterogeneity underlining the clinical variability observed in EO‐PE offspring.

GSEA highlighted an EMT like proteomic signature in the cargo of EO‐PE‐derived sEVs, significant compared to T‐sEVs and PT‐sEVs. Given the endothelial origin of these vesicles and the similarity between EMT and endothelial‐to‐mesenchymal transition (EndoMT), our findings suggest vascular remodelling profiles such as cytoskeletal reorganisation, and the acquisition of mesenchymal characteristics in EO‐PE‐derived sEVs, as observed by others earlier (Schliefsteiner et al. 2024; Wang et al. 2002; Wesseling et al. 2018). Studies report PE associated vascular maladaptation in the foetal compartment, such as reduced umbilical cord lumen size, thickened vessel walls and increased muscularisation (Blanco et al. 2011; Herzog et al. 2017). Reduced endothelial progenitor cells in cord blood (Kwon et al. 2007) and diminished tubule formation and reduced migratory capacity in PE‐derived HUVECs (Brodowski et al. 2017), further suggest vascular dysfunction in PE. Elevated MMP‐9‐ and pro‐inflammatory cytokine levels in cord blood (Galewska et al. 2008) indicate dysregulated extracellular matrix turnover. Importantly, PE‐derived EVs from umbilical cord plasma induce endothelial dysfunction in HUVECs, providing evidence that PE‐derived vesicle cargo may modulate endothelial response in vitro (Ying et al. 2021). As placental sEVs represent a subset of cord plasma derived EVs (Miranda et al. 2018), our data suggest that fpEC derived sEVs, enriched in proteins involved in matrix remodelling and vascular adaptation (e.g., EMT, myogenesis, apical junction and coagulation pathways), may contribute to endothelial activation and altered vascular singling in the foetal compartment.

Beyond the acute vascular changes seen in PE, these alterations may also affect long‐term offspring health. Epidemiological studies consistently report that children born after PE have higher blood pressure, increased arterial stiffness and signs of early vascular aging (Hoodbhoy et al. 2021; Jayet et al. 2010; Lazdam et al. 2010). The EMT‐ and myogenesis‐enriched proteomic signature observed in EO‐PE sEVs may therefore represent early molecular changes that contribute to these long‐term cardiovascular risks.

Our findings align with studies showing that syncytiotrophoblast‐derived EVs in PE reduce eNOS expression, impair endothelial repair and carry anti‐angiogenic proteins like endoglin and sFlt‐1, contributing to PE‐related endothelial dysfunction (Motta‐Mejia et al. 2017; Tannetta et al. 2013). Similarly, PE‐derived EVs enhance vasoconstriction and reduce endothelium‐dependent vasorelaxation in different models (Erlandsson et al. 2023; Murugesan et al. 2022; Powell et al. 2022). We hypothesize that fpEC‐derived sEVs with enriched EMT cargo in PE contribute to vascular maladaptation in the fetoplacental unit and foetus.

To determine the functional role of the EMT‐ and ECM‐related proteomic signature of EO‐PE‐derived sEVs, primary HUVECs were used as a target model in our study. These experiments were designed to capture early endothelial responses rather than definitive mesenchymal transition or long‐term functional effects. EMT and early remodelling processes were assessed by gene expression analysis and brightfield‐based morphological evaluation. Exposure to EO‐PE‐derived sEVs primarily resulted in downregulation of CDH5 (VE‐cadherin), a key component of endothelial junctional destabilisation, indicative of endothelial activation and early remodelling. This finding is consistent with previous reports of reduced VE‐cadherin expression in HUVECs followed by treatment with PE‐derived EVs (Gu et al. 2022; Wu et al. 2023). A similar reduction of CDH5 expression in HUVECs was also observed by PT‐sEVs treatment, suggesting that processes such as stabilisation of cell‐to‐cell contacts and regulation of vascular permeability might not be exclusive to EO‐PE, but may also corelate to PT‐associated placental dysfunction. Interestingly, mesenchymal and EMT‐associated gene expression remained largely unchanged across all treatment conditions, indicating a preservation of the endothelial integrity as described by Dejana et al. (2017). Consistent with this, vWF expression was increased in T‐sEV–treated HUVECs and expression differed significantly between PT‐ and EO‐PE–sEV, suggesting a pregnancy condition dependent changes in endothelial barrier dysfunction without signs to a mesenchymal shift. Morphologically, HUVECs retained a confluent cobblestone monolayer, with only minor changes such as occasional cell elongation in the EO‐PE‐ and PT‐sEV treated HUVECs. This supports an early endothelial response rather than EMT driven phenotype. Importantly, although EO‐PE‐sEVs were enriched in EMT and ECM‐related proteins, associated with mesenchymal cell transition had not progressed beyond the early stages of endothelial activation in the analysed cell culture.

T‐derived sEVs were predominantly enriched with proteins involved in cell cycle control and DNA repair, leading to endothelial dysfunction and potential premature senescence (Tadesse et al. 2014). Elevated circulating factors like HtrA4 and placental oxidative stress can alter EC proliferation and down‐regulate cell‐cycle genes. While some cells show DNA damage markers, responses vary, with some activating the cell cycle rather than senescence, highlighting the complexity of PE pathogenesis (Wang et al. 2019). Increased oxidative stress and impaired proliferation capacity are also observed in cord blood derived from PE pregnancies, indicating these traits extend to the foetal circulation (Baser et al. 2022). PE manifests through functional alternations in cord blood cells, such as hematopoietic stem cells and erythroid cells, with reduced proliferative and differentiation potential (Masoumi et al. 2019; Nordin et al. 2020). As placental EVs are present in cord plasma (Miranda et al. 2018) we hypothesize that placental sEVs interact with cord blood cells, affecting their proliferation and cell cycle capacity. This diminished proliferation has been observed in foetal glomerular endothelial cells (Gu et al. 2023) and HUVECs (Ying et al. 2021) treated with PE‐derived cord plasma EVs. In the maternal system, loss of DNA repair capacity (Rao et al. 2024) and oxidative stress (Baig et al. 2014) have been reported in PE‐derived placental EVs.

A major strength of this study is the use of clinically well‐characterised primary human fpECs from T, PT and EO‐PE pregnancies. The isolation, characterisation and purity of fpEC‐derived sEVs from these groups, compared across protein profiles, make this study unique. Including PT—fpECs is particularly valuable, as it distinguishes gestational age‐dependent effects from those specific to EO‐PE in the EV proteome. Our proteomic analysis, using unsupervised clustering, provides the first evidence that EO‐PE alters the proteomic cargo of placental sEVs secreted into the foetal compartment. A key limitation is the small sample size, particularly for the EO‐PE group, reflecting the rarity of Early‐Onset PE. Additionally, this study focuses on proteomic profiling and does not cover other bioactive EV components, such as microRNAs or lipids, which may contribute to functional differences. While our in vitro data on HUVECs indicate an early endothelial response to fpEC‐derived sEVs, further studies are required to determine whether these early responses may translate to functional consequences on target foetal cells in vitro and in vivo.

Our sEV proteomic approach, based on fpEC‐derived sEVs isolated by differential ultracentrifugation, has some limitations. The sEV‐enriched fraction may contain non‐vesicular proteins, as complete vesicle purity cannot be guaranteed by sequential ultracentrifugation. In addition, proteomics preferentially detects medium‐ to high‐abundance proteins, likely underrepresenting low‐abundance cargo. To address this bias, we focused on functional enrichment across the full protein dataset. Finally, fpEC‐derived sEVs represent only one placental cell source and do not capture contributions from other placental cell types. Despite these limitations, our study reveals proteomic differences between placental sEVs from physiological and pathophysiological pregnancies, offering novel insights into how PE may disrupt foetal cellular function via sEV cargo, its translation and signalling.

## 6 Conclusion

This study provides the first comprehensive characterisation of sEVs secreted by primary fpECs from T‐, PT‐ and EO‐PE pregnancies. Overall, the proteomic cargo is largely conserved, but EO‐PE‐derived sEVs exhibit specific enrichment of proteins involved in endothelial activation and cytoskeletal cell reorganisation, all linked to mechanisms as seen in early EMT. T‐derived sEVs are enriched in cell cycle‐dependent proteins, which suggests that they support proliferative processes more than PT‐ and EO‐PE‐derived sEVs do. Although the focus in PE is primarily on maternal circulation, our use of primary fpECs and proteomic profiling suggests a potential role for fetoplacental EVs in placental‐foetal communication relevant to foetal development. Future studies on cargos and functional validation of sEVs on target cells are needed to elucidate their role in metabolic alterations in offspring after PE pregnancies and their impact on long‐term health.

## Author Contributions

**Michaela Stoiber**: conceptualization, investigation, writing – original draft, methodology, validation, visualization, writing – review and editing, software, formal analysis, data curation, resources. **Monika Horvat Mercnik**: conceptualization, investigation, writing – original draft, methodology, validation, visualization, writing – review and editing. **Birgit Hirschmugl**: conceptualization, validation, visualization, writing – review and editing. **Barbara Darnhofer**: investigation, methodology, writing – review and editing, software, data curation. **Dominique Pernitsch**: investigation, methodology, writing – review and editing, software, data curation. **Barbara Leopold‐posch**: investigation, methodology, writing – review and editing, validation. **Ursula Hiden**: methodology, resources. **Dagmar Kolb**: investigation, conceptualization, writing – review and editing, software. **Christian Wadsack**: conceptualization, funding acquisition, writing – original draft, writing – review and editing, project administration, supervision, resources.

## Ethics Statement

Ethical approval was obtained from the ethics committee of the Medical University of Graz (29‐319 ex 16/17) and detailed voluntary consent was obtained from all study participants.

## Conflicts of Interest

The authors declare no conflicts of interest.

## Supporting information



Supplementary Methods and Supplementary Figures 1–3 are available in following file: Stoiber_et_al_JEXB_Supplements.docx

## Data Availability

The raw mass spectrometry proteomics data have been deposited to the ProteomeXchange Consortium with the dataset identifier: PXD068733.
