# Supplementary material for: Proteomic Epithelial‐To‐Mesenchymal Transition Signature in Fetoplacental Small Extracellular Vesicles of Early‐Onset Preeclampsia
Source: J Extracell Biol. 2026 Mar 3;5(3):e70122. doi: 10.1002/jex2.70122 (PMC12957779; doi:10.1002/jex2.70122)
Supplement: Supplementary file 1 — Supplementary Methods and Supplementary Figures 1–3 are available in following file: Stoiber_et_al_JEXB_Supplements.docx [file JEX2-5-e70122-s001.docx]

Proteomic Epithelial-To-Mesenchymal Transition Signature in Fetoplacental Small Extracellular Vesicles of Early-Onset Preeclampsia

**Michaela Stoiber^1^, Monika Horvat Mercnik^1^, Birgit Hirschmugl^1^, Barbara Darnhofer^2^, Dominique Pernitsch^3^, Barbara Leopold-Posch^1^, Ursula Hiden^1,4^, Dagmar Kolb^3,5^, Christian Wadsack^1,6*^**

^1^ Department of Obstetrics and Gynaecology, Medical University of Graz, Graz, Austria.

^2^ Core Facility Mass Spectrometry, Medical University of Graz, Graz, Austria

^3^ Core Facility Ultrastructure Analysis, Medical University of Graz, Graz, Austria

^4^ Research Unit Early Life Determinants, Medical University of Graz, Graz, Austria

^5^ Gottfried Schatz Research Center for Cell Signaling, Metabolism and Aging, Division of Cell Biology, Histology and Embryology, Medical University of Graz, Neue Stiftingtalstraße 6/II, 8010, Graz, Austria

^6^ BioTechMed-Graz, Graz, Austria

^*^ Corresponding Author

# Supplementary Methods

## Isolation and Culture of Human Umbilical Vein Endothelial Cells (HUVECs)

Human umbilical vein endothelial cells (HUVECs) were isolated from 10-15 cm umbilical cord segments obtained after delivery. Immediately after collection, an intravenous catheter (Venflon, BD, Vienna, Austria) was carefully inserted into the umbilical vein toward the fetal end of the cord and residual blood and clots were removed by repeated flushing with sterile phosphate-buffered saline (PBS). Both ends of the cord were then clamped, and the vein vas filled via the catheter with 10 ml of pre-warmed Collagenase A solution (Roche, Mannheim, Germany). The cord was incubated for 10 minutes at 37 °C. Following enzymatic digestion, the clamp distal to the catheter was released first, and the collagenase effluent containing detached endothelial cells was collected dropwise into a tube, containing 10 ml fetal calf serum (FCS, HyClone, Cytiva, Logan, UT, USA ) to stop enzymatic activity. The proximal clamp was subsequently released, and the vein was gently flushed twice with PBS. All fractions were pooled and centrifuged at 200 × g for 7 minutes at room temperature. The supernatant was aspirated, and the cell pellet was suspended in endothelial ECGM supplemented with endothelial cell growth factor, hydrocortisone, epidermal growth factor, 0.05 mg/mL gentamicin and 5% defined FCS and seeded into a T75 culture flask pre-coated with 1% gelatine (Sigma-Aldrich, Darmstadt, Germany). Cells were cultured at 37 °C in a humidified atmosphere with 21% oxygen. Non-adherent cells were removed by medium change on the following day, and the medium was subsequently replaced every two days.

### Immunocytochemical staining of HUVECs

After the first passage, endothelial identity and purity were confirmed by immunocytochemical staining confirmed by immunocytochemical staining for the endothelial cell markers von Willebrand factor (VWF) and CD31, with complete absence of the smooth muscle cell marker smooth muscle actin (SMA) and the fibroblast marker CD90 as described following: HUVECs (50,000 per 1.7 cm² chamber) were seeded on glass chamber slides (Thermo Scientific Nunc) for 24 h. Cells were then washed with HBSS, air-dried overnight at room temperature (RT), fixed with ice-cold acetone (Merck) for 3 min, and washed again with HBSS. Slides were rehydrated for 3 min in TBE buffer (pH 7.5) containing 0.1% Tween (Sigma, St. Lois, MO, USA), which was also used for all washing steps. Slides were blocked with 3% bovine serum albumin (BSA; Sigma-Aldrich) in 1xPBS for 30 min, followed by washing. Primary antibodies against CD31 (Monosan, Uden, The Netherlands), VWF (Dako, Glostrup, Denmark), SMA (Dako) and CD90 (Dianova, Hamburg, Germany), as well as isotype-matched negative controls (Dako) at identical dilutions (Dako antibody diluent) were applied and incubated for 60 min. Immunodetection was performed using the M/R ImmunoDetector AEC HPR Red Kit (BioSB, Santa Barbara, CA, USA). Briefly, slides were incubated with Link solution for 10 min, washed and incubated with HRP Label. After further washing, the peroxidase chromogen was applied. Finally, slides were rinsed with distilled water, counterstained with hematoxylin, and mounted with Aquatex (both from Sigma-Aldrich).

## Treatment of human umbilical vein endothelial cells (HUVECs) with T-, PT-, and fp-EC derived sEVs

sEVs were isolated from T-, PT-, and PE-fpECs (n=3 per group) CM collected from three 175 cm² flasks (75 mL of CM per group) and pooled. The resulting sEV pellet was resuspended in 250 µL RPMI (Gibco, #11835030, 0.02 µm sterile filtered) and aliquoted into 10 microcentrifuge tubes and stored at -80°C until use. Particle concentration was determined using NTA.

HUVECs were seeded in six-well plates at a density of 2.2 × 10^5^ cells per well and allowed to adhere overnight. On the day of the treatment, cells were serum-starved in ECGM without FCS for 2 hours. Cells were then treated with sEVs at concentrations of 5000 sEVs per cell. Cells cultured in FCS-free medium without sEV treatment, processed in parallel under identical conditions to sEV treated cells were used as control.

Culture medium was replaced after 24 h with fresh ECGM containing the respective treatments. Cells were harvested at timepoint 0 hours (baseline control, untreated) and after 48 hours. For each condition, two wells were used per time point, and cells were pooled during harvesting. Cell culture images were acquired at 4x magnification using an Olympus BX53 brightfield microscope with a SC50 Olympus camera and CellSens software.

## Quantitative Real-Time PCR (RT-qPCR)

Cells were harvested by washing twice with ice-cold HBSS, followed by lysis in 700 μL Qiazol Lysis Reagent (Qiagen, Cat #79306). Total RNA was extracted using the miRNeasy Mini Kit (Qiagen, Cat #217004) according to the manufacturer’s instructions, including on-column DNase digestion with the RNase-Free DNase Set (Qiagen, Cat #79254) to remove genomic DNA contamination. RNA concentration and purity were assessed using the NanoDrop One/Oneᶜ Microvolume UV-Vis Spectrophotometer (Thermo Fisher Scientific). For reverse transcription, 1 μg of RNA was converted into cDNA using the Luna Script RT SuperMix Kit (New England BioLabs, Cat #M3010). Quantitative PCR (qPCR) was performed using the SYBR Green Luna Universal qPCR Master Mix (New England BioLabs, Cat #M3003) on a CFX-384 Touch Real-Time PCR Detection System (Bio-Rad). Gene expression levels were normalized to the housekeeping genes 18S, and HPRT using the -ΔΔCt method.

A list of used primers is provided in Supplementary Table 1.

## Data Analysis

Gene expression levels were calculated using the -ΔΔCt method and are presented to the respective control condition. Statistical analysis were performed on the ΔCt values using GraphPad Prism (version 8.4.3). Data were tested for normality using the Shapiro-Wilk test. Outliers were identified using the ROUT method. For normally distributed data, paired one-way ANOVA followed by Tukey’s multiple-comparisons test was applied. If normality was not met, data were analysed using the Friedman test with Dunn’s multiple-comparisons test. Data were presented in a heatmap showing -ΔΔCt generated in RStudio using ComplexHeatmap package in R.

# Supplementary figures


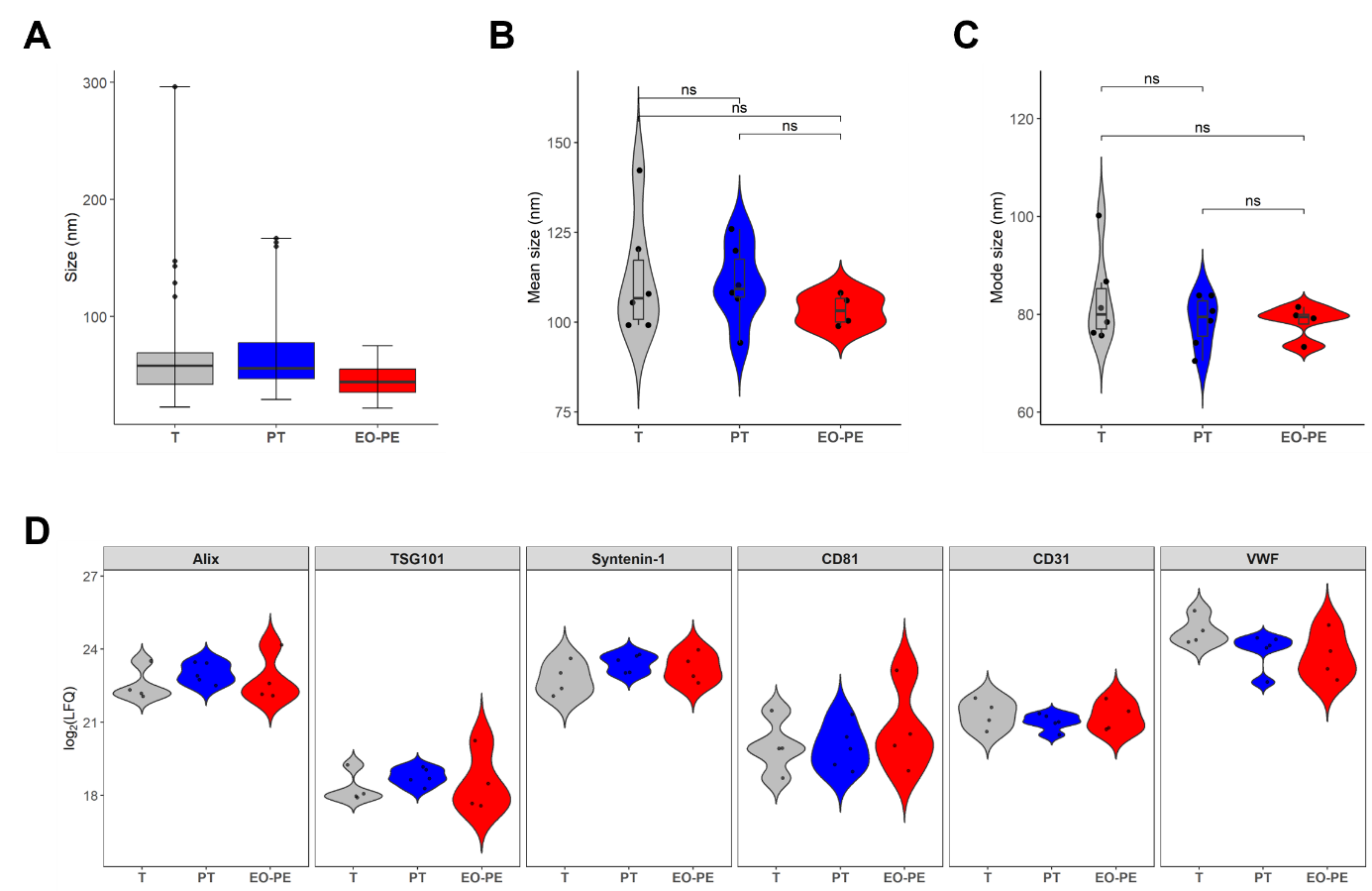


**Supplementary Figure 1:** Additional information for the characteristics of fpECs. A) quantitative size analysis of sEVs (n=50 vesicles per group) of TEM images. B) Mean size evaluated by NTA C) Mode size evaluated by NTA. Statistical analysis was performed using the Kruskal–Wallis test, followed by pairwise Wilcoxon rank-sum tests with Bonferroni correction for multiple comparisons. D) Log_2_ transformed LFQ values of Alix, TSG101, Syntenin-1, CD81, CD31 and VWF from the proteomic analysis validating the presence of endothelial and EV-markers in sEVs


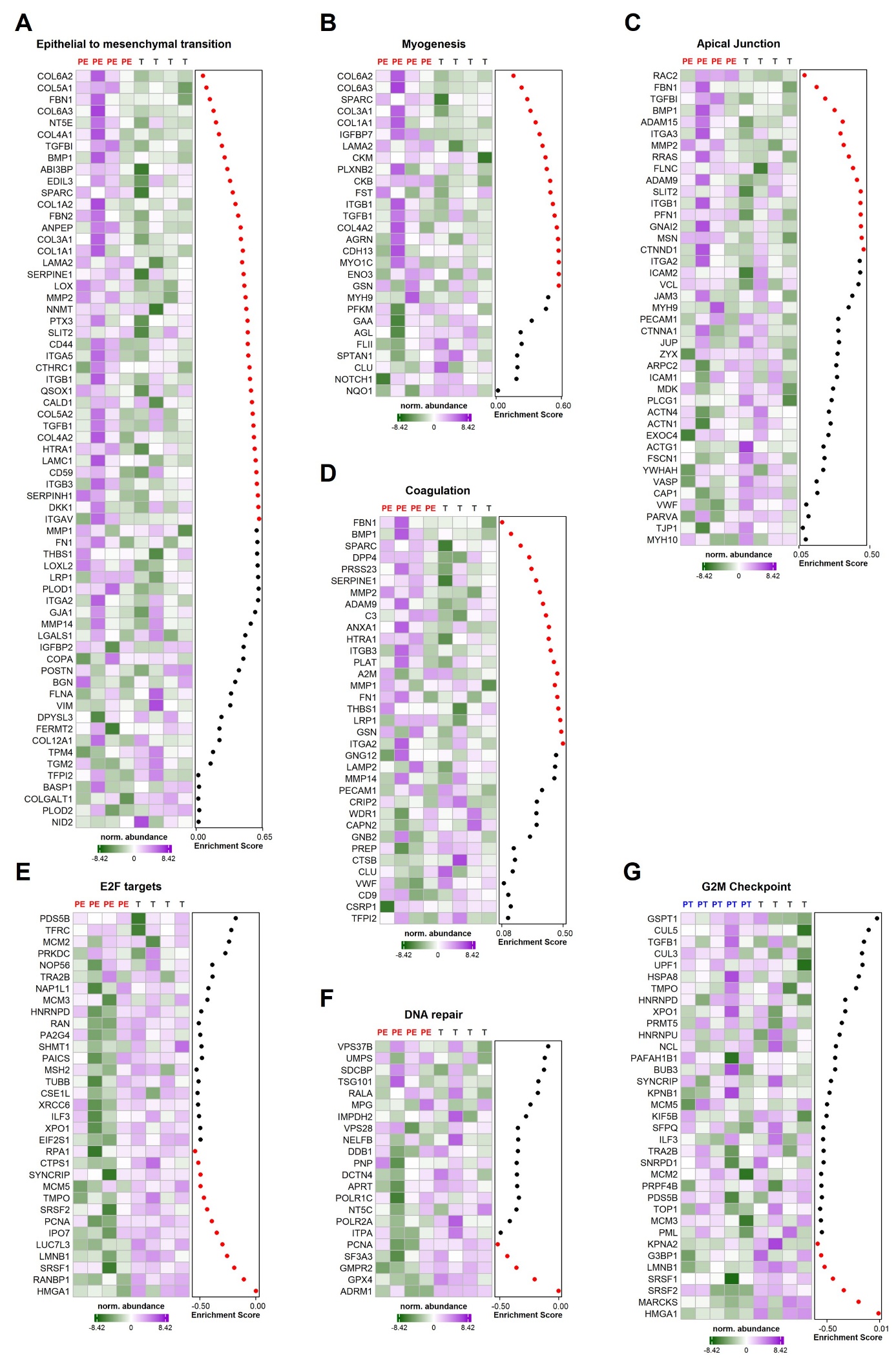


**Supplementary Figure 2**: Heatmaps show the normalized abundance of individual proteins within the representative enriched pathways in A) Epithelial-to-mesenchymal transition (EMT, EO-PE vs. T), B) Myogenesis (EO-PE vs. T), C) Apical Junction (EO-PE vs. T), D) Coagulation (EO-PE vs. T), E) E2F targets (EO-PE vs. T), F) DNA repair (EO-PE vs. T) and G) G2M Checkpoint (PT vs. T). Each row represents a protein, ranked by their contribution to pathway enrichment. The enrichment score for each protein is shown to the right of each heatmap. Core-enriched proteins are highlighted in red, while non-core-enriched proteins are shown in black.


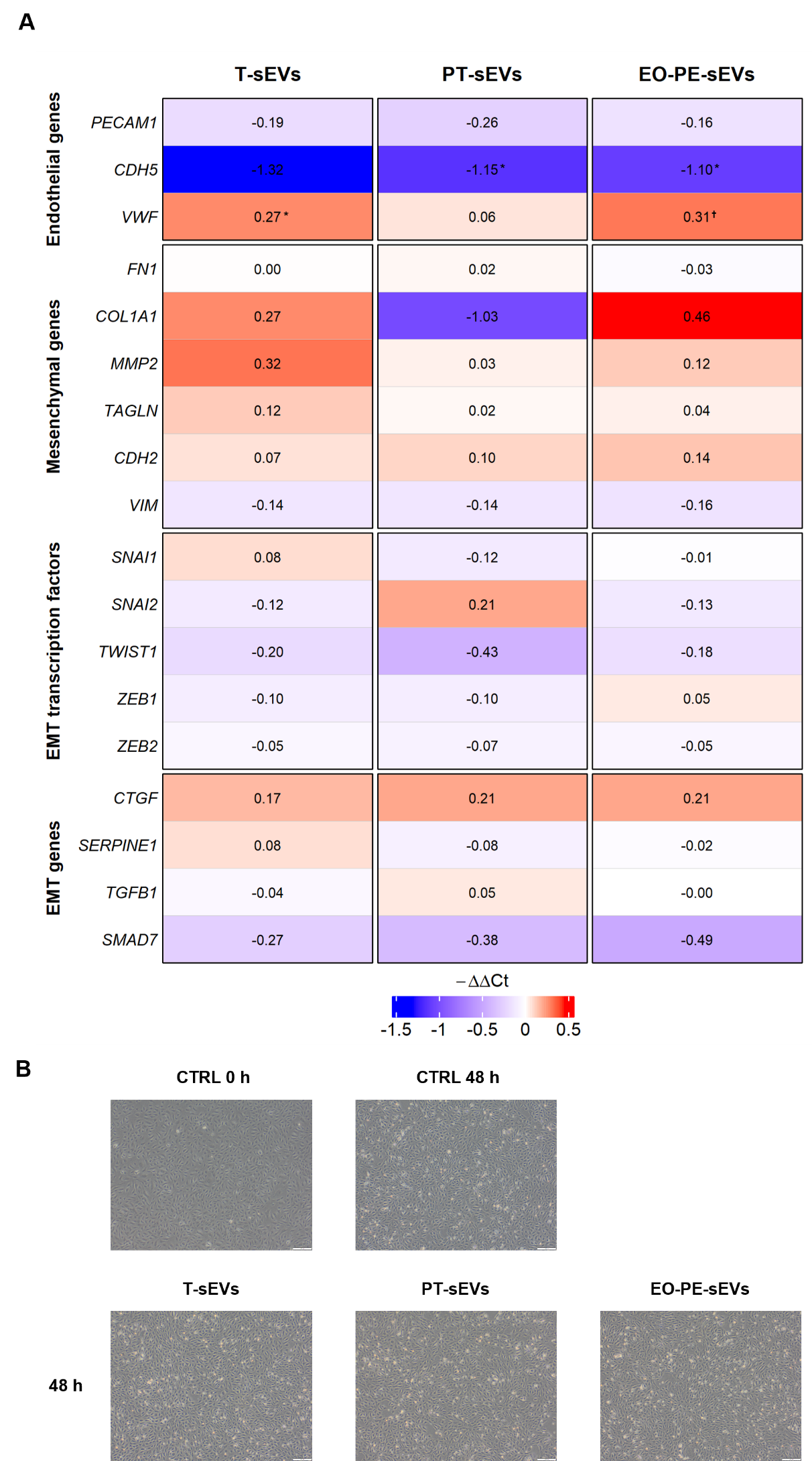


**Supplementary Figure 3: A)** Heatmap presenting average -ΔΔCt values (normalized to untreated CTRL) of primary HUVECs treated with 5000 sEVs derived from T-, PT- and EO-PE fpECs. Statistical analyses were performed on ΔCt using either paired One-way ANOVA followed by Tukey’s posthoc test or Friedman test followed by Dunn’s posthoc. *p < 0.05 indicates statistical significance (CTRL vs. PT, CTRL vs. EO-PE) and ^†^p < 0.05 indicates statistical significance (PT vs. EO-PE). B) Representative brightfield images of HUVEC at baseline (0 h) and after 48 h of treatment with 5000 sEVs derived from T-, PT- and EO-PE fpECs. CTRL indicates cells cultured in FCS-free medium without sEV treatment. Images were taken at 4x magnification, scale bar represents 200µm.

# Supplementary Table

Supplementary Table 1: RT-qPCR primer list used in this study. The table includes gene name, supplier information and forward (F) and reverse primer sequences (5`→3`). For targets quantified using Qiagen QuantiTect Primer Assays, assay names and GeneGlobe identifiers are provided.

| **Gene name** | **Supplier** | **Primer (F/R)** |
| --- | --- | --- |
| *18S rRNA* | Merck | F(5`→3`): CTACCACATCCAAGGAAGCA R(5`→3`): TTTTTCGTCACTACCTCCCCG |
| *CDH2* | Merck | F(5`→3`): CAGACCGACCCAAACAGCAAC  R(5`→3`): GCAGCAACAGTAAGGACAAACATC |
| *CDH5* | Merck | F(5`→3`): TCGTTGCGCTCTTCGTGAC  R(5`→3`): CAGCCCGCAAAACAGGTAG |
| *COL1A1* | Merck | F(5`→3`): AAAGGCAATGCTCAAACACC R(5`→3`): TCAAAAACGAAGGGGAGATG |
| *CTFG* | Merck | F(5`→3`): TTGCGAAGCTGACCTGGAAGAGAA R(5`→3`): AGCTCGGTATGTCTTCATGCTGGT |
| *FN1* | Merck | F(5`→3`): CGTCATAGTGGAGGCACTGA R(5`→3`): CAGACATTCGTTCCCACTCA |
| *HPRT1* | Qiagen | Hs_HPRT1_1_SG QuantiTect Primer Assay GeneGlobe ID: QT00059066\|Cat. No.: 249900 |
| *MMP2* | Qiagen | Hs_MMP2_1_SG QuantiTect Primer Assay GeneGlobe ID: QT00088396\|Cat. No.: 249900 |
| *PECAM1* | Merck | F(5`→3`): ATCGGTTGTTCAATGCGTCC R(5`→3`): CCTTCAGGATTTGGTACATGACA |
| *SERPINE1* | Merck | F(5`→3`): CACAAATCAGACGGCAGCACT  R(5`→3`): CATCGGGCGTGGTGAACTC |
| *SMAD7* | Merck | F(5`→3`): TCCAGATGCTGTGCCTTCC  R(5`→3`): GTCCGAATTGAGCTGTCCG |
| *SNAI1* | Merck | F(5`→3`): TCGGACCCACACATTACCTT R(5`→3`): TGAGCCCTCAGATTTGACCT |
| *SNAI2* | Merck | F(5`→3`): CCAGTGCCTCGACCACTATG R(5`→3`): CTGCTGGAAGGTAAACTCTGGA |
| *Tangl* | Merck | F(5`→3`): TTCAAGCAGATGGAGCAGGT R(5`→3`): TGCCATGTCTTTGCCTTCAA |
| *TGFB1* | Merck | F(5`→3`): AACCCACAACGAAATCTAATG R(5`→3`): CTTTTAACTTGAGCCTCAGC |
| *TWIST1* | Merck | F(5`→3`): GGAGTCCGCAGTCTTACGAG R(5`→3`): CTGGAGGACCTGGTAGAGG |
| *VIM* | Merck | F(5`→3`): TGGCACGTCTTGACCTTGAA R(5`→3`): AGCTCCTGGATTTCCTCTTCG |
| *VWF* | Merck | F(5`→3`): AGTGCTGTGATGAGTATGAGTG R(5`→3`): GATGGTGCTTCGGTGGAC |
| *ZEB1* | Merck | F(5`→3`): AGCAGTGAAAGAGAAGGGAATGC R(5`→3`): GGTCCTCTTCAGGTGCCTCA |
| *ZEB2* | Merck | F(5`→3`): CACAAGCCAGGGACAGATCA R(5`→3`): TTGCCACACTCTGTGCATTTG |
